# Effect of Three Different Commonly Available Gingival Retraction Systems on the Amount of Gingival Displacement: An In Vivo Study

**DOI:** 10.7759/cureus.106510

**Published:** 2026-04-06

**Authors:** Pamei Jenthuilu, Sandeep Kumar, Rajnish Aggarwal, Rahul Sharma, Navneet S Kathuria, Paras Kumar, Mansi Sharma

**Affiliations:** 1 Department of Prosthodontics, Surendera Dental College and Research Institute, Sri Ganganagar, IND; 2 Department of Oral and Maxillofacial Surgery, Dr. S.S. Tantia Dental College, Hospital and Research Centre, Sri Ganganagar, IND; 3 Department of Oral Pathology and Microbiology, Dr. S.S. Tantia Dental College, Hospital and Research Centre, Sri Ganganagar, IND

**Keywords:** displacement, gel, gingival, paste, retraction cord

## Abstract

Introduction: Accurate recording of finish line margins is essential for the success of fixed prosthodontic restoration. Gingival displacement plays a crucial role in achieving adequate exposure of the subgingival margins, thereby ensuring proper marginal adaptation and long-term periodontal health. Various gingival retraction systems, including conventional cords and newer cordless materials such as pastes and gels, are available; however, their comparative effectiveness remains a topic of clinical interest. This study aimed to evaluate and compare the effectiveness of three commonly used gingival retraction systems in achieving gingival displacement based on pre- and post-retraction measurements.

Materials and methods: This prospective observational in vivo study included 15 systemically healthy participants aged 18-25 years old. Gingival displacement was evaluated on the maxillary right central incisor using three retraction systems: retraction cord (n = 15), astringent retraction paste (n = 15), and Racegel (n = 15). The control group (n = 15) had no gingival retraction. Baseline impressions were recorded without retraction, followed by post-retraction impressions after the application of each system under standardized conditions. Thus, each participant underwent four impression procedures, yielding a total of 60 impressions. One impression without gingival retraction served as the control, while three additional impressions were recorded following gingival displacement using the respective retraction systems. Consequently, 15 control impressions and 45 impressions after gingival retraction (15 per system) were obtained. The casts were sectioned and analyzed using a stereomicroscope and ImageJ software (National Institutes of Health (NIH), Bethesda, MD, USA). Horizontal and vertical displacements were measured in micrometers (µm). Statistical analyses were performed using one-way analysis of variance and post-hoc Tukey's tests.

Results: The mean horizontal displacement was highest in the retraction cord group (92.289 ± 28.724 µm), followed by Racegel (62.409 ± 21.978 µm) and retraction paste (58.213 ± 18.485 µm), with statistically significant differences among the groups (p < 0.001). Similarly, vertical displacement was greatest with retraction cord (458.530 ± 112.414 µm), followed by retraction paste (416.638 ± 110.911 µm) and Racegel (370.697 ± 68.476 µm), with significant differences (p < 0.001).

Conclusion: The retraction cord demonstrated superior gingival displacement compared to the cordless systems. However, Racegel and retraction paste provided clinically acceptable results and may be considered alternatives in situations prioritizing patient comfort and minimal tissue trauma.

## Introduction

In contemporary fixed prosthodontics, the success of indirect restorations largely depends on the precision of impression making, particularly the accurate recording of the preparation margins [1]. Subgingival or equigingival finish lines, often required for esthetic or functional reasons, pose significant challenges because of interference from gingival tissues, crevicular fluid, and occasional hemorrhage. Effective gingival displacement is a critical step that allows the impression material to adequately capture the finish line, ensure marginal integrity, and contribute to long-term periodontal health and restoration longevity [2,3].

Gingival displacement is defined as the controlled lateral and vertical movement of the marginal gingiva away from the prepared tooth surface. A minimum sulcular width of 0.15-0.20 mm is generally considered necessary to achieve void-free, dimensionally stable impressions and prevent tearing or distortion of the impression material [4]. Inadequate displacement frequently results in incomplete marginal reproduction, poor adaptation, secondary caries, gingival inflammation, and eventual restoration failure [4].

Over the decades, numerous gingival retraction techniques have evolved, ranging from purely mechanical methods (retraction cords) to chemomechanical systems (medicated cords or injectable pastes/gels) and surgical approaches (electrosurgery and lasers) [5]. Among these, gingival retraction cords remain the most widely used because of their proven effectiveness, affordability, and ability to provide predictable horizontal and vertical displacement, especially when impregnated with hemostatic/astringent agents such as aluminum chloride [6,7]. However, cord placement is often associated with patient discomfort, increased chair time, gingival trauma, and the risk of biologic width violation if improperly performed.

To overcome these limitations, cordless retraction systems, including pastes and gels, have gained popularity. These systems offer rapid application, reduced tissue trauma, better patient acceptance, and effective hemostasis through a combination of chemical and gentle mechanical actions [8,9]. Despite their growing clinical use, conflicting evidence exists regarding their comparative efficacy in achieving adequate gingival displacement when compared with conventional cord techniques.

The aim of the present prospective observational in vivo study was to evaluate and compare the effectiveness of three commonly used gingival retraction systems, astringent retraction paste, retraction cord, and Racegel, in achieving gingival displacement based on pre- and post-retraction measurements. This study sought to quantitatively assess horizontal and vertical gingival displacement by measuring changes in sulcus dimensions before and after application of each retraction system under standardized clinical conditions. Furthermore, this study aimed to compare the mean displacement produced by each system to determine their relative efficacy in facilitating adequate gingival retraction for accurate impression making in fixed prosthodontics.

## Materials and methods

The present study was designed as a prospective observational in vivo study, where randomization, allocation concealment, or blinding was not performed. All clinical procedures were carried out in the Department of Prosthodontics at Surendera Dental College and Research Institute, Sri Ganganagar, Rajasthan, India, over a period of six months from January 2025 to June 2025. Ethical approval for this study was obtained from the Institutional Ethics Committee of the college (Reference No.: SDCRI/IEC/D/24/25; dated 18/03/24). The study protocol adhered to the ethical principles outlined in the Declaration of Helsinki (2013). Written informed consent was obtained from all participants after a detailed explanation of the study objectives, procedures, potential risks, and benefits.

A total of 15 systemically healthy participants aged 18-25 years were recruited based on predefined inclusion and exclusion criteria. Participants included in the study had an intact, unrestored maxillary right central incisor with clinically healthy gingival and periodontal tissues (Plaque Index = 0 and Gingival Index = 0). Individuals with gingival or periodontal disease, systemic illness, a history of medication affecting gingival health, smoking habits, pregnancy or lactation, or prior prosthodontic treatment or trauma in the maxillary anterior region were excluded from the study.

For standardization, only the maxillary right central incisor of each participant was selected for the evaluation. An initial baseline impression without gingival retraction was recorded using addition silicone (polyvinyl siloxane) impression material with a custom tray fabricated using self-curing acrylic resin and a double-layer spacer technique. The impressions were poured into the type IV die stone to obtain baseline casts.

Each participant underwent four impression procedures, yielding a total of 60 impressions. One impression without gingival retraction served as the control, while three additional impressions were recorded following gingival displacement using the respective retraction systems. Consequently, 15 control impressions and 45 impressions after gingival retraction (15 per system) were obtained. Sufficient time between procedures was given to permit gingival tissue recovery and minimize any carryover effects. The three systems evaluated were 3M ESPE Astringent Retraction Paste (3M ESPE, Seefeld, Germany) (n = 15), SureCord Plus retraction cord impregnated with aluminum chloride (Sure Endo, Goyang-si, South Korea) (n = 15), and Racegel (Septodont, Saint-Maur-des-Fossés, France) (n = 15). In the control group (n = 15), no gingival retraction was performed. The astringent retraction paste was applied directly into the gingival sulcus using a dispensing capsule and left in place for 3 min. The retraction cord was gently packed into the sulcus using a cord-packing instrument and left for 5 min. Racegel was injected into the sulcus using the manufacturer-provided applicator tip and left for 2 min. Following the specified application time, the materials were carefully removed, and the sulcus was rinsed and gently air-dried.

A standardized washout period of 48 hours was maintained between successive procedures to allow adequate gingival tissue recovery and to minimize carryover effects; gingival health was reassessed prior to each procedure to ensure return to baseline conditions. The sequence of application of the retraction systems was randomized using a simple randomization method to reduce order bias. All clinical procedures were performed by a single experienced operator to ensure consistency, while image analysis was carried out by the same examiner who was blinded to the study groups.

Immediately after the removal of each retraction system, a post-retraction impression was recorded using the same addition silicone material and technique. All impressions were disinfected using 2% glutaraldehyde and poured with type IV die stone. The resulting casts were sectioned labiopalatally through the center of the maxillary right central incisor to obtain approximately 3 mm-thick sections using a die-cutting machine.

**Figure 1:**
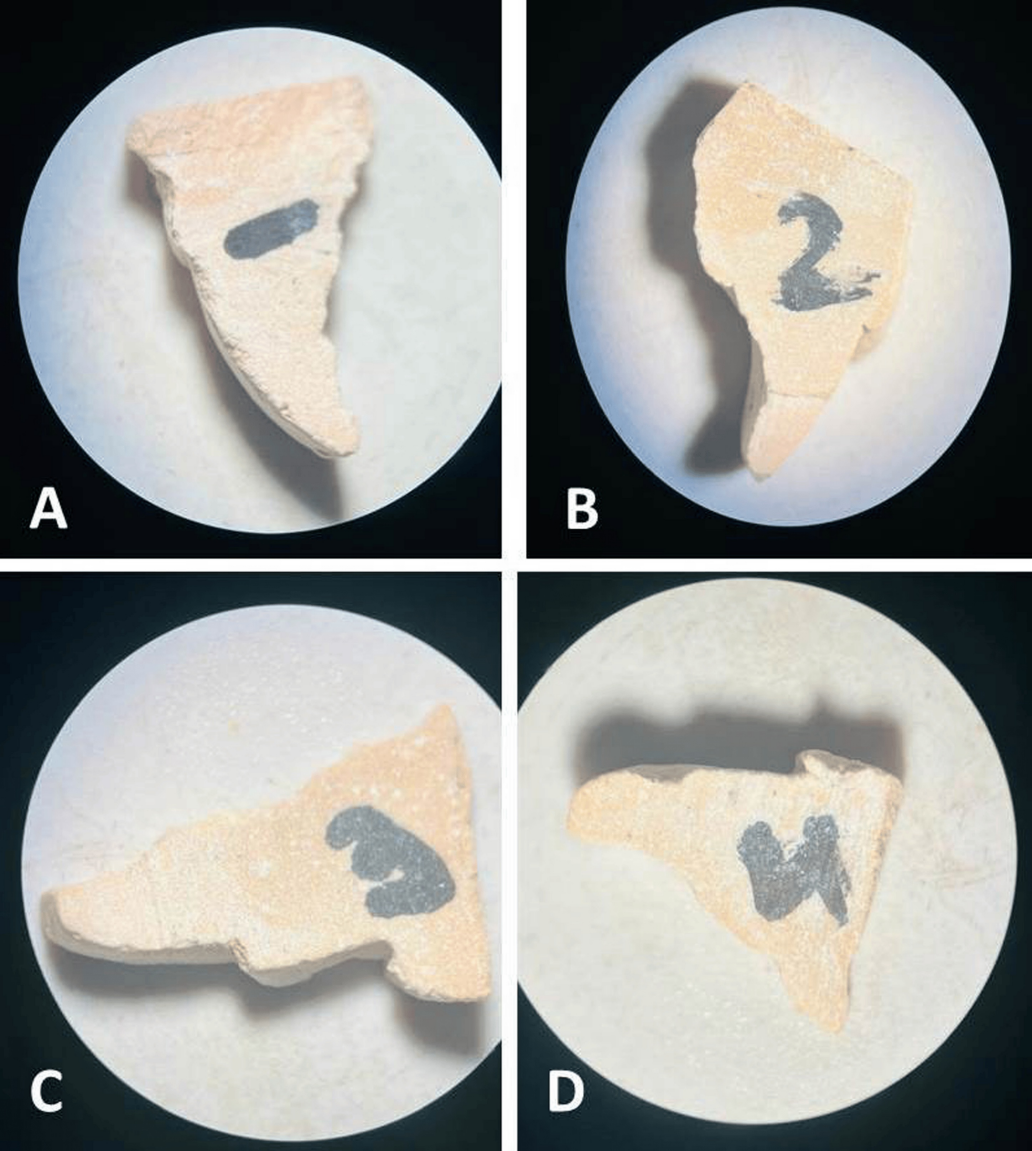
Stereomicroscopic evaluation (20× magnification) of sectioned casts illustrating gingival displacement: (A) control group (no gingival retraction); (B) specimens treated with 3M ESPE Astringent Retraction Paste; (C) specimens treated with SureCord Plus retraction cord; and (D) specimens treated with Racegel.

The sectioned samples were examined using a stereomicroscope at 20× magnification (Figure 1). The obtained images were analyzed using ImageJ software (National Institutes of Health (NIH), Bethesda, MD, USA), which was calibrated using a stage micrometer prior to measurement to ensure accuracy in micrometer (µm) units. Horizontal gingival displacement was measured as the perpendicular distance from the tooth surface to the crest of the gingival margin, and vertical gingival displacement was measured as the distance from the gingival margin to the base of the sulcus. Gingival displacement was calculated by subtracting the baseline (pre-retraction) values from the post-retraction values and expressed in micrometers (µm). Each measurement was performed three times, and the mean value was used for analysis to minimize intra-examiner error. The sample size was calculated using G*Power software (version 3.1) (Heinrich-Heine-Universität, Düsseldorf, Germany), assuming a medium effect size, with a power of 80% and a significance level (α) of 0.05; although the minimum required sample size was lower, 15 participants (60 impressions) were included to improve the reliability of the findings.

All recorded data were compiled and analyzed statistically. Descriptive statistics, including the mean and standard deviation, were calculated for both horizontal and vertical gingival displacement. The normality of the data distribution was assessed using the Shapiro-Wilk test. As the same participants were evaluated across all three conditions, intergroup comparisons were performed using one-way analysis of variance (ANOVA), followed by post-hoc Tukey's test for pairwise comparisons. Statistical significance was set at p < 0.05.

## Results

Fifteen participants were included in this study. The mean age of the participants was 20.7 ± 3.2 years, consisting of 8 (53.3%) males and 7 (46.7%) females. A comparison of horizontal gingival displacement among the study groups is shown in Table 1. The mean horizontal displacement was highest in the retraction cord group (92.289 ± 28.724 µm), followed by Racegel (62.409 ± 21.978 µm) and retraction paste (58.213 ± 18.485 µm), whereas the control group showed the least displacement (46.896 ± 16.883 µm). The differences among the groups were statistically significant (F = 11.631, p < 0.001).

**Table 1:** Comparison of horizontal displacement among study groups. *p < 0.05 denotes results were statistically significant with one-way analysis of variance.

Groups	n	Mean (µm)	Standard deviation	F value	p-value
Control group	15	46.896	16.883	11.631	<0.001*
Retraction paste	15	58.213	18.485		
Retraction cord	15	92.289	28.724		
Racegel	15	62.409	21.978		

The pairwise comparisons of the horizontal displacements are presented in Table 2. A statistically significant difference was observed between the control group and retraction cord (p < 0.001), retraction paste and retraction cord (p < 0.001), and retraction cord and Racegel (p = 0.003). No statistically significant differences were found between the control group and retraction paste (p = 0.499), the control group and Racegel (p = 0.227), or retraction paste and Racegel (p = 0.953).

**Table 2:** Pairwise comparisons of horizontal displacement among study groups. *p < 0.05 denotes results were statistically significant with post-hoc Tukey's test.

Pairwise group comparison	Mean difference (µm)	p-value
Control group vs. retraction paste	-11.317	0.499
Control group vs. retraction cord	-45.393	<0.001*
Control group vs. Racegel	-15.513	0.227
Retraction paste vs. retraction cord	-34.076	<0.001*
Retraction paste vs. Racegel	-4.196	0.953
Retraction cord vs. Racegel	29.880	0.003*

A comparison of vertical gingival displacement among the study groups is shown in Table 3. The highest mean vertical displacement was observed in the retraction cord group (458.530 ± 112.414 µm), followed by retraction paste (416.638 ± 110.911 µm) and Racegel (370.697 ± 68.476 µm), whereas the control group exhibited the lowest displacement (261.559 ± 90.153 µm). The differences among the groups were statistically significant (F = 11.434, p < 0.001).

**Table 3:** Comparison of vertical displacement among study groups. *p < 0.05 denotes results were statistically significant with one-way analysis of variance.

Groups	n	Mean (µm)	Standard deviation	F value	p-value
Control group	15	261.559	90.153	11.434	<0.001*
Retraction paste	15	416.638	110.911		
Retraction cord	15	458.530	112.414		
Racegel	15	370.697	68.476		

The pairwise comparisons of the vertical displacements are summarized in Table 4. Statistically significant differences were observed between the control group and all three retraction systems: retraction paste (p < 0.001), retraction cord (p < 0.001), and Racegel (p = 0.017). No statistically significant differences were observed between the retraction paste and cord (p = 0.641), retraction paste and Racegel (p = 0.570), or retraction cord and Racegel (p = 0.075).

**Table 4:** Pairwise comparisons of vertical displacement among study groups. *p < 0.05 denotes results were statistically significant with post-hoc Tukey's test.

Pairwise group comparison	Mean difference (µm)	p-value
Control group vs. retraction paste	-155.079	<0.001*
Control group vs. retraction cord	-196.971	<0.001*
Control group vs. Racegel	-109.137	0.017*
Retraction paste vs. retraction cord	-41.892	0.641
Retraction paste vs. Racegel	45.941	0.570
Retraction cord vs. Racegel	87.833	0.075

## Discussion

The findings of our study demonstrated that all three systems produced measurable gingival displacement; however, the magnitude of displacement varied significantly among them, with the retraction cord exhibiting the highest efficacy, followed by Racegel and the retraction paste. The superior performance of the retraction cord observed in this study can be attributed to its combined mechanical and chemical actions. When impregnated with aluminum chloride, the cord not only physically displaces the gingival tissues but also induces transient shrinkage of the gingival tissues and controls sulcular fluid through its astringent effect. This dual mechanism allows for greater lateral and apical displacement of the gingiva, thereby enhancing the sulcular width and depth [6]. These findings are consistent with those of previous studies that reported that conventional retraction cords provide greater gingival displacement than cordless systems. For instance, Gupta et al. and Gajbhiye et al. demonstrated that cord-based systems achieved significantly higher horizontal displacement owing to their ability to exert sustained mechanical pressure within the sulcus [10,11].

In contrast, cordless systems, such as astringent retraction paste and Racegel, rely primarily on chemical action with minimal mechanical force. The relatively lower displacement observed with the retraction paste in the present study may be due to its limited ability to exert sufficient pressure within the sulcus, particularly in cases with thicker gingival biotypes. Although these materials are easier to apply and more comfortable for patients, their efficacy in achieving adequate sulcular widening may be compromised compared to that of traditional cord techniques. This observation aligns with the findings of Rajbanshi et al., who reported that retraction pastes provide less displacement but are associated with reduced gingival trauma and improved patient comfort [12]. In contrast, another study reported better retraction with retraction paste than with the cord. This disparity could be due to the different materials used [13].

Racegel demonstrated intermediate performance between the retraction cord and paste systems. Its gel-based formulation allows better flow into the sulcus and provides a mild mechanical effect, in addition to its chemical astringency. The relatively higher displacement observed with Racegel compared to that with retraction paste may be attributed to its viscosity and ability to maintain contact with the sulcular walls, thereby enhancing tissue displacement. The findings of the present study are consistent with those of a systematic review by Huang et al., which compared cord- and cordless-based gingival retraction techniques [14]. The authors reported that although cordless systems, such as pastes and gels, offer advantages in patient comfort, hemostasis, and reduced tissue trauma, their ability to achieve gingival displacement is often inferior to that of conventional retraction cords, particularly at subgingival margins. Furthermore, the review highlighted conflicting evidence regarding the magnitude of displacement achieved by cordless systems, suggesting that both techniques are clinically effective but may be indicated in different scenarios.

The findings of the present study are partially consistent with those reported by Dawood and Majeed, who conducted an in vivo comparative evaluation of different gingival retraction materials, including Racegel and astringent retraction paste [15]. Their study demonstrated statistically significant differences in gingival displacement among the tested materials, with astringent retraction paste producing the highest displacement and Racegel exhibiting the least effectiveness. These findings support the results of the present study, where gel-based systems showed comparatively lower gingival displacement than conventional methods. The reduced efficacy of Racegel may be attributed to its limited mechanical action and reliance primarily on chemical astringency, which may be insufficient to achieve adequate sulcular widening, particularly in the deeper sulci.

The results of the present study also highlighted that all three retraction systems produced significantly greater vertical displacement than the control (no retraction), indicating their effectiveness in exposing the finish line for impression making. Vertical displacement is particularly important for capturing subgingival margins and ensuring the marginal accuracy of restorations. The significantly higher vertical displacement observed with the retraction cord further reinforces its role as the gold standard, especially in cases requiring deep subgingival margin recording.

Despite the superior performance of retraction cords, their clinical use is often associated with certain disadvantages, including technique sensitivity, increased chairside time, and potential for gingival trauma or bleeding if improperly placed. In contrast, cordless systems offer advantages such as ease of application, reduced patient discomfort, and minimal tissue injury. Therefore, while cord systems may be preferred in situations requiring maximum displacement, cordless systems may be considered in cases with a shallow sulcus, thin gingival biotype, or when patient comfort is a priority.

The findings of this study are clinically relevant, as achieving adequate gingival displacement is essential for accurate impression making and the long-term success of fixed prosthodontic restorations. Insufficient displacement can result in marginal discrepancies, leading to microleakage, secondary caries, and periodontal complications. The present study reinforces the importance of selecting an appropriate gingival retraction system based on clinical requirements, balancing efficacy, patient comfort, and tissue preservation.

The present study had certain limitations. The sample size was relatively small, which may limit the generalizability of our findings. This study evaluated only immediate gingival displacement, and long-term effects, such as gingival rebound and tissue recovery, were not assessed. Additionally, the absence of randomization and blinding may have introduced potential bias. The study also did not simulate intraoral conditions, such as saliva, blood contamination, and functional loading, which could influence the effectiveness of retraction systems in clinical practice.

## Conclusions

Within the limitations of this prospective observational in vivo study, all three gingival retraction systems demonstrated the ability to produce a measurable gingival displacement. However, the retraction cord exhibited significantly greater horizontal and vertical displacement than Racegel and astringent retraction paste, indicating its superior efficacy in achieving adequate sulcular widening for precise impression making. Among the cordless systems, Racegel and retraction paste showed comparatively lower but clinically acceptable displacements. Therefore, although the retraction cord remains the gold standard, cordless systems may serve as suitable alternatives in situations requiring minimal tissue trauma and improved patient comfort.

## References

[1] Cooper LF (2009). The current and future treatment of edentulism. J Prosthodont.

[2] Alraheam IA, Hattar S, Al-Asmar A, Alhadidi A, Hamour SA, Aldroubi A, Sawair FA (2023). Dentists' knowledge and preference regarding gingival displacement methods. BMC Oral Health.

[3] Madaan R, Paliwal J, Sharma V, Meena KK, Dadarwal A, Kumar R (2022). Comparative evaluation of the clinical efficacy of four different gingival retraction systems: an in vivo study. Cureus.

[4] Safari S, Ma VS, Mi VS, Hamedi M (2016). Gingival retraction methods for fabrication of fixed partial denture: literature review. J Dent Biomater.

[5] Tabassum S, Adnan S, Khan FR (2017). Gingival retraction methods: a systematic review. J Prosthodont.

[6] Kavita K, Sinha RI, Singh R, Singh R, Reddy KR, Kulkarni G (2020). Assessment of aluminum chloride retraction cords, expasyl, and tetrahydrozoline-soaked retraction systems in gingival retraction. J Pharm Bioallied Sci.

[7] Makakova DR, Zagorchev P, Dimitrova M, Georgieva Y, Tilov B (2024). Absorptive capacity of gingival retraction cords in hemostatic solutions: an in vitro study. Medicina (Kaunas).

[8] Mehta S, Virani H, Memon S, Nirmal N (2019). A comparative evaluation of efficacy of gingival retraction using polyvinyl siloxane foam retraction system, vinyl polysiloxane paste retraction system, and copper wire reinforced retraction cord in endodontically treated teeth: an in vivo study. Contemp Clin Dent.

[9] Shaw DH, Krejci RF, Cohen DM (1980). Retraction cords with aluminum chloride: effect on the gingiva. Oper Dent.

[10] Gupta A, Prithviraj DR, Gupta D, Shruti DP (2013). Clinical evaluation of three new gingival retraction systems: a research report. J Indian Prosthodont Soc.

[11] Gajbhiye V, Banerjee R, Jaiswal P, Chandak A, Radke U (2019). Comparative evaluation of three gingival displacement materials for efficacy in tissue management and dimensional accuracy. J Indian Prosthodont Soc.

[12] Rajbanshi MK, Kinra MS, Sharanesha RB, Virupakshappa D, Khojah AB, Almakenzi AA, Almakenzi SA (2025). Efficacy of different gingival displacement materials on the width of gingival sulcus using optical stereomicroscope: a comparative analysis. J Pharm Bioallied Sci.

[13] Rathod A, Jacob SS, MAlqahtani A, Valsan I, Majeed R, Premnath A (2021). Efficacy of different gingival displacement materials in the management of gingival sulcus width: a comparative study. J Contemp Dent Pract.

[14] Huang C, Somar M, Li K, Mohadeb JV (2017). Efficiency of cordless versus cord techniques of gingival retraction: a systematic review. J Prosthodont.

[15] Dawood ZM, Majeed MA (2015). An evaluation of the efficacy of different gingival retraction materials on the gingival tissue displacement (a comparative in vivo study). J Baghdad Coll Dent.

